# An evolutionary perspective on Y-chromosomal variation and male infertility

**DOI:** 10.1111/j.1365-2605.2008.00889.x

**Published:** 2008-08

**Authors:** Chris Tyler-Smith

**Affiliations:** The Wellcome Trust Sanger Institute, Wellcome Trust Genome CampusCambridgeshire, UK

**Keywords:** fertility, infertility, single nucleotide polymorphism, spermatogenic failure, structural variation, Y chromosome

## Abstract

Genetic variation on the Y chromosome is one of the best-documented causes of male infertility, but the genes responsible have still not been identified. This review discusses how an evolutionary perspective may help with interpretation of the data available and suggest novel approaches to identify key genes. Comparison with the chimpanzee Y chromosome indicates that *USP9Y* is dispensable in apes, but that multiple copies of *TSPY1* may have an important role. Comparisons between infertile and control groups in search of genetic susceptibility factors are more complex for the Y chromosome than for the rest of the genome because of population stratification and require unusual levels of confirmation. But the extreme population stratification exhibited by the Y also allows populations particularly suitable for some studies to be identified, such as the partial *AZFc* deletions common in Northern European populations where further dissection of this complex structural region would be facilitated.

## Introduction

Why should andrologists take an interest in evolution? A simple answer is that evolution can be seen as fertility averaged over a long period of time, and thus may highlight subtle aspects of fertility that might not be obvious in a single generation. Fertility varies between individuals because of both biological and social factors. Among the biological factors are genetic ones; indeed mutations in some 10% of mouse genes can lead to infertility (Kile *et al.*, 2003) and the proportion is likely to be similar in humans, although the identity of most of these genes is unknown. An evolutionary perspective provides expectations about them. If a gene is functionally important, we expect it to be evolutionarily conserved and thus present in related species. Conversely, if a candidate infertility gene is not conserved, we might question its importance for fertility, and will discuss an example of such a gene below. Over the shorter period of evolution within human populations, we might expect to see that variants which increase fertility increase in frequency in the population, whereas variants that reduce fertility decrease in frequency. These simple expectations are, however, affected by the complete linkage over most of the Y chromosome: selection acts on the whole haplotype. Thus, a disadvantageous mutation on an otherwise beneficial haplotype background can still increase in frequency and even reach fixation, whereas an advantageous mutation on a disadvantageous background might still be selected against.

Why take a special interest in the Y chromosome? Genes on the Y have received a large and perhaps disproportionate amount of attention because their relevance has been appreciated for more than 30 years (Tiepolo & Zuffardi, 1976), and they remain as the largest known genetic cause of male infertility. The male-specific region of the Y chromosome reference sequence codes for 27 proteins (Skaletsky *et al.*, 2003), and we might expect that several of these would be required for fertility. Three classical regions where deletions can lead to azoospermia have been identified (Vogt *et al.*, 1996), pointing to the presence of genes involved in spermatogenesis, but considerable uncertainty still surrounds the number and identity of the genes underlying the *AZF* phenotypes.

Why write another review on the Y chromosome and infertility? The topic deserves special attention because of the unique properties of the Y, discussed in more detail below, which can result in its omission from standard reviews or genomic surveys of genes relevant to fertility (e.g. Lessard *et al.*, 2004). Thus, it has been the subject of periodic focussed reviews, with one as recently as 2006 (Krausz & Degl’Innocenti, 2006). Since then, the sequence of much of the Y chromosome of our closest living relative, the chimpanzee *Pan troglodytes*, has been published (Hughes *et al.*, 2005; Kuroki *et al.*, 2006), there have been substantial advances in our understanding of structural variation in the Y chromosomes of normal humans (Redon *et al.*, 2006; Repping *et al.*, 2006; Jobling *et al.*, 2007), and new studies of how Y variation relates to spermatogenesis or fertility have been reported (Arredi *et al.*, 2007; Lu *et al.*, 2007; Vodicka *et al.*, 2007; Yang *et al.*, 2008). This article will review our current knowledge of Y variation, how patterns of variation might point to differences in fertility, and how the unique population genetics of the Y chromosome can both undoubtedly hinder but perhaps also help the search for causal variants.

## Y-chromosomal variants

Variants may directly influence fertility, but they can also be used indirectly by marking lineages: there is complete linkage over most of the length of the Y chromosome. Variants can conveniently be considered within three categories: (i) SNPs (single nucleotide polymorphisms or base substitutions; sometimes used to include binary markers of many types including small insertions and deletions), (ii) STRs (short tandem repeats or microsatellites; multiallelic) and (iii) structural variants, of which CNVs (copy number variants; binary or multiallelic) have received most attention. Genome databases such as Ensembl (http://www.ensembl.org/Homo_sapiens/index.html) now list very large numbers of Y-SNPs: 72 220 in January 2008. Many of these, however, may represent differences between a Y sequence and a related sequence elsewhere in the genome (paralogs), and less than 1% are SNPs that have been placed on a phylogenetic tree (Jobling & Tyler-Smith, 2003). Databases of Y-SNP phylogeny are available (e.g. http://www.snp-y.org/) and there are plans to construct databases summarizing geographical distributions. Y-STRs can be identified from the reference sequence and a comprehensive survey identified 475 potentially useful loci (Kayser *et al.*, 2004); these are the Y markers of choice for forensic work and comprehensive databases containing over 52 000 haplotypes exist (Willuweit & Roewer, 2007), but they have been less used in infertility studies.

**Figure 1:**
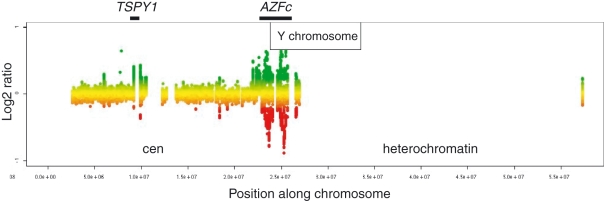
Normal copy number variation on the human Y chromosome. Log2 ratios from comparative genomic hybridization to BAC clones spanning the euchromatic portion of the Y chromosome were superimposed from 269 HapMap individuals (reproduced from Redon *et al.*, 2006 supplementary figure 6). Regions of the Y chromosome showing the most copy number variation are visualized as green and red segments above and below the yellow line. The *TSPY1* and *AZFc* regions are the most copy number variable (top); the largest two gaps correspond to the centromere and Yq heterochromatin (bottom).

The extent of structural variation in the general population is only just beginning to be appreciated (Freeman *et al.*, 2006), but it is now apparent that this form of variation affects more nucleotides per individual than SNP variation (Redon *et al.*, 2006). No substantial region of the genome is free of structural variation, but a genomewide perspective shows that the Y is particularly enriched, with the *AZFc* and *TSPY* regions standing out most (Fig. 1; Redon *et al.*, 2006). More focussed surveys have also emphasized the Y’s high level of structural variation (Repping *et al.*, 2006) and the geographical specificity of some significant variants: for example, chromosomes lacking *AMELY*, *TBL1Y* and *PRKY* are present at a frequency of ∼2% among normal men in the Indian subcontinent but rare elsewhere (Jobling *et al.*, 2007).

Much information on Y-chromosomal variation is thus available, and the emerging genomewide resequencing of individual genomes (e.g. Levy *et al.*, 2007) is planned to expand to 1000 genomes over the next 3 years (http://www.1000genomes.org/index.html), half of which should be male and carry Y chromosomes. Thus, the normal range of variation on the Y is becoming increasingly well documented. Further requirements are for databases that provide information on the geographical distributions of Y-SNPs and Y-structural variants, and most of all for better functional understanding of the variants.

## The evolutionary fate of variants

Most variants have no detectable effect on the phenotype or fitness, and so are considered neutral. The evolutionary fate of a new neutral mutation depends on chance (genetic drift) and is influenced by characteristics of the population such as its size, including whether it is expanding or contracting, and the amount of exchange (migration) with other populations: the fields of population and evolutionary genetics (Jobling *et al.*, 2004). In contrast, beneficial variants will be positively selected and tend to increase in frequency, whereas harmful variants will be negatively selected and decrease in frequency. In the most extreme case, for example, a variant that leads to complete spermatogenic failure, the variant will not be transmitted and will be found in the population at the level determined by mutation.

Two conclusions from theoretical and empirical studies of neutral or near-neutral variants are particularly relevant to the current review. First, the effective population size of the Y chromosome is low: there are one-quarter of the number of Y chromosomes in the population compared with any autosome, and the large variance in male offspring numbers reduces this even further, making the Y more susceptible to drift than any other locus. The prevalence of patrilocality, whereby children tend to be born near the father’s birthplace rather than the mother’s, increases the geographical clustering of Y variants. So Y chromosomes differ more between different places than any other part of the genome. Second, departures from neutral expectation, such as an unusually rapid increase in a Y lineage, can indicate selection and are of particular interest.

The abundance of data on Y variants described in the last section has allowed some lineages that have expanded very rapidly – exhibited very high fertility – to be identified: one each in Central (Zerjal *et al.*, 2003) and East Asia (Xue *et al.*, 2005) and one in Europe (Moore *et al.*, 2006). In all cases, this high fertility could be explained by social rather than biological factors: the Mongol emperor Genghis Khan, the Chinese emperor Nurhaci or the Irish dynasty Neill, respectively. Comprehensive worldwide surveys of Y variation now in progress (e.g. The Genographic Project https://www3.nationalgeographic.com/genographic/index.html) will reveal how common such selective events are, and whether any may represent new biological variants that increase fertility, rather than powerful male-line dynasties.

## Perils of population stratification

If we want to know whether or not a genetic variant influences a phenotype of interest, e.g. sperm count, a standard approach is to measure the frequency of the variant in samples of individuals who differ in the phenotype. If we found, say, 40% A allele in the men with high sperm count and 70% in the men with low sperm count, we might want to conclude that the A allele marked a genetic background that led to low sperm count. But we should be very cautious before coming to this conclusion: the two samples might differ for other reasons, for example, if they come from different geographical regions. This is known as ‘population stratification’. It is the important characteristic that makes the Y chromosome so popular for evolutionary studies, noted above, but it also makes association studies involving the Y chromosome fraught with difficulty. The magnitude of this effect is illustrated by a paper published in 1999 which investigated the association between Y haplogroup and infertility in Italy (Previderé*et al.*, 1999). In the raw data, haplogroup P was present at 42% in the controls but 24% in the infertile men, a statistically significant difference. But the infertile men were mostly sampled in Central Italy, whereas the controls were from several parts of Italy. When only Central Italians were considered, the frequencies were 27 and 26% respectively, a non-significant difference. This is a far greater degree of geographical differentiation than detected with ∼10 000 autosomal SNPs (Bauchet *et al.*, 2007), rendering ineffective one of the recommended methods of correcting for stratification, genomic control. How then can the careful investigator of Y-chromosomal associations produce reliable results? Precise geographical matching is essential and replication in an independent sample is also necessary.

Many studies have sought to identify Y-chromosomal influences on spermatogenic failure, sperm count or male infertility. Some have taken the approach of comparing haplogroups between relevant samples and reported no effect (Paracchini *et al.*, 2002; Lu *et al.*, 2007) or significant differences (e.g. Kuroki *et al.*, 1999; Krausz *et al.*, 2001; Yang *et al.*, 2008), although such differences have not always been replicated (Carvalho *et al.*, 2003). Others have compared Y variants that alter the gene content, particularly partial deletions of the *AZFc* region (Repping *et al.*, 2003 and many subsequent studies), a complex field that will be covered in another minireview in this series.

Another set of studies has investigated the effect of haplogroup background on microdeletion frequency. Here, the microdeletions themselves almost always lead to spermatogenic failure; the question is whether or not the mutations that produce such deletions occur at different rates on different lineage backgrounds. Studies using pooled samples from several European regions (Paracchini *et al.*, 2000; Quintana-Murci *et al.*, 2001) or Israel (Carvalho *et al.*, 2004) detected no effect; but a study that carefully matched controls and deletions from the same part of Italy (Arredi *et al.*, 2007) found an increased susceptibility to *AZFc* microdeletion in one lineage, haplogroup E, while a study of samples from Sichuan (Southwest China) reported an increased frequency in O3* (Yang *et al.*, 2008). These investigations therefore meet one of the criteria for demonstrating an effect, precise geographical matching, but now need to be replicated in independent samples.

## Benefits of population stratification

**Figure 2:**
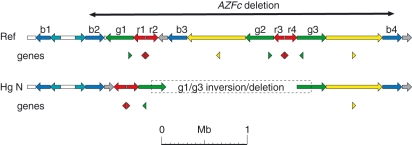
Making use of population stratification. In the reference Y chromosome sequence (Ref), nine genes are present in the *AZFc* region belonging to three gene families with two, three and four copies each. In contrast, haplogroup N Y chromosomes (Hg N) show normal spermatogenesis but are partially deleted, with only four genes present from the same three gene families, with one, one and two copies each. Thus, populations where haplogroup N Y chromosomes are common, such as those in Northern Europe, would be suitable for searching for point mutations associated with spermatogenic failure in these genes.

While population stratification is generally a confounding influence for association studies, it may be possible to take advantage of it some circumstances. Although Y microdeletions are indisputably associated with spermatogenic failure, the roles of the individual genes lost remain unclear. The microdeletions all remove multiple genes, but it should still be possible to identify the key ones by searching for *de novo* point mutations in single genes. Yet such an approach has not been very successful, and a major reason for this is the repeated nature of the *AZFb* and *AZFc* deleted regions: the genes they contain are present in multiple copies and inactivation of a single copy may not lead to spermatogenic failure. The b2/b4 complete *AZFc* deletion removes nine genes including all members of the three families *BPY2*, *DAZ* and *CDY1* (Fig. 2; Kuroda-Kawaguchi *et al.*, 2001), almost always resulting in spermatogenic failure. In contrast, the g1/g3 (=b2/b3) partial deletion removes five genes leaving one *BPY2*, two *DAZ* and one *CDY1* (Fernandes *et al.*, 2004; Repping *et al.*, 2004; Fig. 2), but is found in haplogroup N men with normal spermatogenesis who make up half the population of northern Europe (Zerjal *et al.*, 1997, 2001). In this relatively simple genetic background, a survey of haplogroup N men with spermatogenic failure might reveal point mutations in the remaining single-copy genes.

## Pointers to Y-chromosomal gene function from evolutionary comparisons between species

Although the *AZFa* microdeletion has the simplest structure and is the best understood of the Y microdeletions, carrying just two genes, *USP9Y* and *DDX3Y* (formerly *DBY*), there has still been debate about which gene/s is/are responsible for spermatogenic failure. A complete deletion of *USP9Y* was associated with severe oligospermia (Brown *et al.*, 1998) whereas a 4-bp deletion in a splice site leading to truncation of the protein was found in an azoospermic man (Sun *et al.*, 1999), suggesting that this gene is required for spermatogenesis. In contrast, two different partial deletions were found in men with moderate oligoasthenoteratozoospermia from families where these Y chromosomes were successfully transmitted under natural conditions (Krausz *et al.*, 2006). It is therefore interesting that the two chimpanzee Y chromosomes sequenced both carry inactive forms of *USP9Y* (Hughes *et al.*, 2005; Kuroki *et al.*, 2006; Tyler-Smith *et al.*, 2006) and the four inactivating mutations in this gene are shared by bonobos (Perry *et al.*, 2007) indicating fertility among apes for perhaps two million years in the absence of *USP9Y*. Such observations point to a role for *DDX3Y* in human spermatogenesis and suggest that further studies of this gene are warranted.

**Figure 3:**
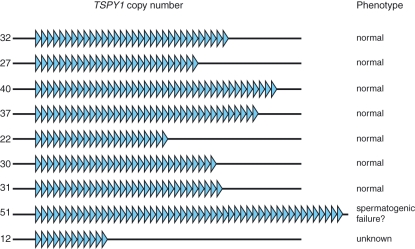
Pointers to gene function from evolutionary considerations. The *TSPY1* gene cluster ranges from about 20–40 copies in normal individuals, and must vary more widely as well. What phenotypes might be associated with larger or smaller numbers? One report suggested an association between increased copy number and infertility (Vodicka *et al.*, 2007), but the phenotype resulting from decreased copy number is unknown and merits further investigation.

The *TSPY1* gene cluster provides one of the few examples of a tandemly repeated protein-coding gene in the human genome. Tandem arrays tend to expand and contract over evolutionary timescales by non-allelic homologous recombination, so it is not surprising that *TSPY1* copy number varies between ∼20–40 copies in the general population (Fig. 3; Tyler-Smith *et al.*, 1988). In the absence of selection for multiple copies of the gene, such variation would eventually lead to the fixation of a single copy, with loss of the other copies. Although there is some uncertainty about the copy number in other apes, the presence of multiple copies (Muller, 1987; Schempp *et al.*, 1995) suggests selection for these additional copies, and thus a disadvantageous phenotype associated with low copy number in humans. One study reported an *increased TSPY1* copy number in infertile patients (Vodicka *et al.*, 2007), but the phenotype associated with *decreased* copy number is unknown and would be an interesting direction for future research.

## Conclusions

Information on Y-chromosomal variation in fertile and infertile men is being generated at an ever-increasing rate. Physiological, cellular and molecular studies can be complemented by an evolutionary perspective to produce increased insights into the genes and mechanisms underlying the complex phenotype of fertility.
